# An ADAM17-Neutralizing Antibody Reduces Inflammation and Mortality While Increasing Viral Burden in a COVID-19 Mouse Model

**DOI:** 10.3389/fimmu.2022.918881

**Published:** 2022-06-10

**Authors:** Jodi F. Hedges, Deann T. Snyder, Amanda Robison, Heather M. Grifka-Walk, Karlin Blackwell, Kelly Shepardson, Douglas Kominsky, Agnieszka Rynda-Apple, Bruce Walcheck, Mark A. Jutila

**Affiliations:** ^1^ Department of Microbiology and Cell Biology, Montana State University, Bozeman, MT, United States; ^2^ Department of Veterinary and Biomedical Sciences, University of Minnesota, St. Paul, MN, United States

**Keywords:** ADAM19, COVID-19, SARS-CoV-2, lung, inflammation, virus, mouse model

## Abstract

Angiotensin Converting Enzyme 2 (ACE2) is the primary cell entry receptor for SARS-CoV and SARS-CoV-2 viruses. A disintegrin and metalloproteinase 17 (ADAM17) is a protease that cleaves ectodomains of transmembrane proteins, including that of ACE2 and the proinflammatory cytokine TNF-α, from cell surfaces upon cellular activation. We hypothesized that blockade of ADAM17 activity would alter COVID-19 pathogenesis. To assess this pathway, we blocked the function of ADAM17 using the monoclonal antibody MEDI3622 in the K18-hACE2 transgenic mouse model of COVID-19. Antibody-treated mice were healthier, less moribund, and had significantly lower lung pathology than saline-treated mice. However, the viral burden in the lungs of MEDI3622-treated mice was significantly increased. Thus, ADAM17 appears to have a critical anti-viral role, but also may promote inflammatory damage. Since the inflammatory cascade is ultimately the reason for adverse outcomes in COVID-19 patients, there may be a therapeutic application for the MEDI3622 antibody.

## Introduction

The COVID-19 pandemic caused by SARS-CoV-2 has caused 500M confirmed infections and more than 6.1M deaths worldwide at the time of this writing. There have been over 81M confirmed cases and one million deaths in the US alone (https://covid.cdc.gov/covid-data-tracker/#datatracker-home). The pandemic continues, due to repeated emergence of viral variants. Fortunately, multiple effective vaccines for COVID-19 have been developed and are in use worldwide. However, variable vaccine efficacy against new variants, lack of vaccine response in some people, and poor public compliance in vaccination underscore the importance of continued development of new therapeutics to complement the vaccine efforts.

SARS-CoV-2 is a positive-sense, single-stranded RNA β-coronavirus that likely arose in bats before spilling over to infect humans, initially in China (1). The virus is a pulmonary pathogen, causing relatively mild disease response in most people. In some patients, infection can induce robust lung inflammation, which can develop into acute respiratory distress syndrome (ARDS), mild to severe pneumonia, and potentially death (2–4). SARS-CoV-2 can also spread systemically and affect multiple organs, such as the liver (5), heart and cardiovascular system (6), central nervous system (7), and gastrointestinal tract (8).

The SARS-CoV-2 spike glycoprotein must bind to host Angiotensin Converting Enzyme 2 (ACE2) expressed at epithelial surfaces prior to infection. Following SARS-CoV-2 binding to ACE2, the viral spike protein is modified by the host protease TMPRSS2, facilitating entry into the epithelial cell and viral replication (9). ACE2 is a dimeric, zinc-dependent metalloprotease that cleaves several substrates. Most prominently, ACE2 converts vasoconstrictive and pro-inflammatory angiotensin II into protective Ang- (1–7), thus ACE2 is an important vascular regulatory component in the Renin-Angiotensin-System (RAS) (10, 11). Through these functions, ACE2 has been shown to be involved in several diseases including COVID-19 (12), acute lung injury (13, 14), heart disease (15), liver and lung fibrosis (16), inflammatory lung disease (17), and cardiopulmonary disease (18).

ADAM17 [a disintegrin and metalloproteinase 17, formerly known as TNF-α-converting enzyme (TACE)] is a transmembrane protease constitutively expressed by most cells, including leukocytes (19, 20). As a membrane sheddase, ADAM17 removes the ectodomain of up to 80 different surface glycoproteins with diverse functions including cytokines, cytokine and growth factor receptors, and adhesion proteins (19, 20). Cell activation by various stimuli rapidly induces ADAM17 proteolytic activity, increasing its intrinsic activity through conformational changes and intermolecular interactions (19). Substrate cleavage by ADAM17 typically occurs in a cis manner at an extracellular site at the cell membrane (19). Excessive and prolonged induction of ADAM17 contributes to various inflammatory disorders (19, 21), presumably through shedding of important inflammatory mediators, such as tumor necrosis factor alpha (TNF-α) and IL-6R (20). Supporting an important role in promoting excessive inflammation induced by infection, inhibition of ADAM17 significantly improves survival during polymicrobial sepsis due to reduced inflammation-associated tissue damage and leukocyte paralysis (22).

ADAM17 is intimately involved in the regulation of ACE2 expression and, potentially, SARS-CoV-2 infection. ADAM17 is activated following SARS-CoV-spike protein binding to ACE2 (23). In addition, ADAM17 is the primary protease that cleaves ACE2 to release its ectodomain, but TMPRSS2 and ADAM10 can also cleave ACE2 (24–27). Internalization during virus entry and proteolytic shedding of ACE2 causes an imbalance in RAS, leading to thrombosis and inflammation (28, 29). These events lead to further activation of ADAM17, amplifying the inflammatory response and likely setting the stage for the cytokine storm and ARDS seen in some COVID-19 patients (2–4). Along these lines, a recent study using a small molecule inhibitor of ADAM17, and other matrix metalloproteases (MMP) showed that the sheddase may be involved in the pulmonary inflammatory response and lung damage induced by polyinosinic:polycytidylic acid and SARS-CoV-2 spike protein in mice (30). Because ADAM17 alters the expression of surface ACE2 and the generation of soluble ACE2 (sACE2), its activity likely affects SARS-CoV-2 virus infection. In support of this, a recent study employing cell culture models showed that blocking ADAM17 enzymatic activity greatly reduced viral replication due to diminished sACE2 (31). In contrast, Zipeto et al. proposes in a review article that ADAM17 activation could dampen viral infection. For instance, ADAM17-generated sACE2 ectodomain within extracellular spaces and the vascular system may bind free virus and prevent attachment to host cells (23). The loss of ACE2 from epithelial cell surfaces due to ADAM17 sheddase activity may also further dampen viral spread. Hence, the ADAM17 sheddase may have distinct roles in SARS-CoV-2 infection and pathogenesis of COVID-19. Understanding the impact of ADAM17 on COVID-19 progression *in vivo* is paramount to understanding the pathophysiological mechanisms underlying the disease and the development of new therapeutics. Here, we show for the first time the impact of ADAM17 on both disease outcome and viral burden in K18-hACE2 mice infected with SARS-CoV-2. Mice treated with an ADAM17-specific monoclonal antibody (MEDI3622) displayed less morbidity than saline-treated mice, appeared healthier, and lost significantly less weight than the saline-treated group. Antibody treated mice displayed significantly less inflammatory damage in the lung compared to saline-treated mice. However, MEDI3622-treated mice had significantly higher viral burdens in lungs. MEDI3622 treatment markedly reduced the levels of TNF-α in bronchoalveolar lavage fluids (BALF), whether it was induced by LPS treatment or by SARS-CoV-2 infection. Thus, induction of ADAM17 activity appears to have a critical anti-viral role, but also may promote damaging inflammation. Since the inflammatory cascade is ultimately the reason for the adverse outcomes in COVID-19 and many other lung inflammatory conditions, there may be a therapeutic application for MEDI3622.

## Materials and Methods

### Mice and Infection

All animal studies were reviewed and approved by the Montana State University IACUC committee. All experiments with SARS-CoV-2 were performed under BSL3 containment. All mice were purchased from the Jackson Laboratory. K18-hACE2 transgenic mice ([B6.Cg-Tg(K18-ACE2)2Prlmn/J, Stock No: 034860] express human ACE2 (hACE2), the receptor used by SARS coronaviruses to gain cellular entry. The K18 promoter directs expression to epithelia, including airway epithelia where infections typically begin. SARS-CoV-2 (WA/01 strain, BEI Resources) was propagated on Vero E6 cells for 3 passages as previously described (32) and quantified by plaque assay. Early passage of this strain was sequenced and deletion in the furin cleavage site known to promote viral infection was detected. However, the virus preparation readily infected and caused disease in K18-ACE2 mice. Mice (eight to eleven weeks old, groups of 8-10) were lightly anesthetized with isoflurane and oxygen, and given 10^4^ PFU SARS-CoV-2 in 30µl by the intranasal route. We had previously determined that this infectious dose induced disease that mimics moderate to severe COVID-19 in humans (data not shown). On day one post-infection, mice were treated intraperitoneally with either 15mg/kg MEDI3622 mAb (MEDI3622) or an equal volume of saline vehicle. Dosing of MEDI3622 was based on similar studies involving a mouse sepsis model (22). Mice were weighed and their health was scored daily on a scale of 0-3 (3=moribund, 20% weight loss; 2 = 15% weight loss, AND hunched posture, possible build-up around eyes, impaired mobility, matted fur; 1 = 10% weight loss, slight (if any) impaired mobility; 0=no symptoms, no weight loss). Mice were euthanized by pentobarbital injection followed by exsanguination on either day 4 or day 6 post-infection. Bronchoalveolar lavage fluids (BALF) and blood were collected from mice euthanized at day 4 post infection. At day 6 post-infection, we collected blood and whole lungs for histology, RNA extraction and virus quantification.

K18-ACE2 mice were also treated with either MEDI3622 or saline and then 1μg *E. coli* LPS by the intraperitoneal and intratracheal routes to artificially induce inflammation. Twenty-four hours later, mice were euthanized and BALF collected for assessment of TNF-α in the lungs.

### Viral Quantification (qPCR/Plaque Assay)

For mice euthanized at day 6 post infection, all portions of the lung besides the left lobe were homogenized in 2ml of HBSS. Following homogenization, 1ml was centrifuged at 500xg for 5 minutes, and the cell pellet frozen at -80 degrees C. After thawing, the pellet was resuspended in 350ul buffer RLT (QIAmp Viral RNA Mini Kit 52906) and RLT-lysed lung homogenate was heat inactivated for at least 20 minutes at 65 degrees C to allow safe removal from BSL3 containment. Homogenized lung lysate was processed through a QIAshredder column and RNA was extracted using the QIAGEN RNeasy Mini kit. RT-qPCR was performed with SARS-CoV-2 (2019-nCoV) CDC RUO Primers and Probes purchased from IDT and Quantabio UltraPlex 1-Step ToughMix RT-qPCR mix.

Virus in lung homogenate supernatant fluids and BALF was measured by plaque assay as previously described (32). Briefly, fluids were 10-fold serially diluted out to 10^-5^ and 300μl was applied to confluent monolayers of low passage Vero E6 cells in a 6 well flat-bottomed plate. The plates were incubated for 1 hour with occasional gentle rocking, then overlayed with 1% methylcellulose in complete DMEM medium. The plates were incubated for 4 days, then the supernatant fluids were removed, and the cell monolayers were stained with 0.5% methylene blue in 70% ethanol to reveal plaques. Plaques were counted and PFU/ml calculated.

### Transcript Analysis

Lung RNA was extracted as described above. RNA was reverse-transcribed using the iScript cDNA Synthesis kit (Bio-Rad 1708891) and gene expression was measured using AzuraQuant Green Fast qPCR Mix (Azura Genomics AZ-2105) and primers targeting genes of interest. Primer sequences are as follows: *ifnb* 5’-AAGAGTTACACTGCCTTTGCCATC-3’ (forward) and 5’-CACTGTCTGCTGGTGGAGTTCATC-3’ (reverse); *ccl5* 5’-GGGTACCATGAAGATCTCTGCA-3’ (forward) and 5’TTGGCGGTTCCTTCGAGTGA-3’ (reverse); *il6* 5’-TAGTCCTTCCTACCCCAATTTCC-3’ (forward) 5’-TTGGTCCTTAGCCACTCCTTC-3’ (reverse); *tnfa 5’-*AAGCCTGTAGCCCACGTCGTA-3’ (forward) 5’- GGCACCACTAGTTGGTTGTCTTTG-3’ (reverse); *il10* 5’-GCTCTTACTGACTGGCATGAG-3’ (forward) and 5’-CGCAGCTCTAGGAGCATGTG-3 (reverse) (33).

### TNF-α Detection

TNF-α was measured in BALF from mice treated with MEDI3622 or saline following either LPS treatment or 4 days after SARS-CoV-2 infection. Mouse TNF-α in BALF from LPS-treated mice was measured using ELISA MAX™ Deluxe Set Mouse TNF-α (Biolegend). TNF-α in BALF collected day 4 post-infection from SARS-CoV-2 infected mice was measured using the highly sensitive ProQuantum Mouse TNF alpha Immunoassay Kit (Invitrogen). We utilized the ProQuantum Assay with BSL-3 samples due to facility restrictions precluding ELISAs.

### Histology

The left lobe of the lung was collected at sacrifice and fixed in 10% formalin, paraffin embedded, sectioned into 5µm sections, and stained with hematoxylin and eosin (H&E) following the manufacturer’s instructions. Histological sections were scored based on a 0-3 scale (0: none; 1: mild; 2: moderate; 3: severe) for seven separate parameters: infiltration of cells (primarily macrophages) across the parenchyma, assessed at 4X power, bronchitis, peribronchitis, vasculitis, perivasculitis, proteinaceous exudate, and edema. Images were acquired using a DS-Ri-1 camera (Nikon, Tokyo, Japan) mounted on a Nikon Eclipse 80i microscope at 4x magnification and analyzed by a histotechnician.

### Statistical Analyses

Statistical analyses were performed using Prism (GraphPad Software, San Diego, CA, USA). The Kolmogorov-Smirnov test (with Dallal-Wilkinson-Liliefor p-value) was used to determine if the data formed a Gaussian distribution. When the data were found to be normally distributed, the two groups were compared using the unpaired students t-test or two-way ANOVA. Otherwise, groups were compared using the Mann-Whitney test. Data were considered significant when p<0.05.

## Results

**Figure 1:**
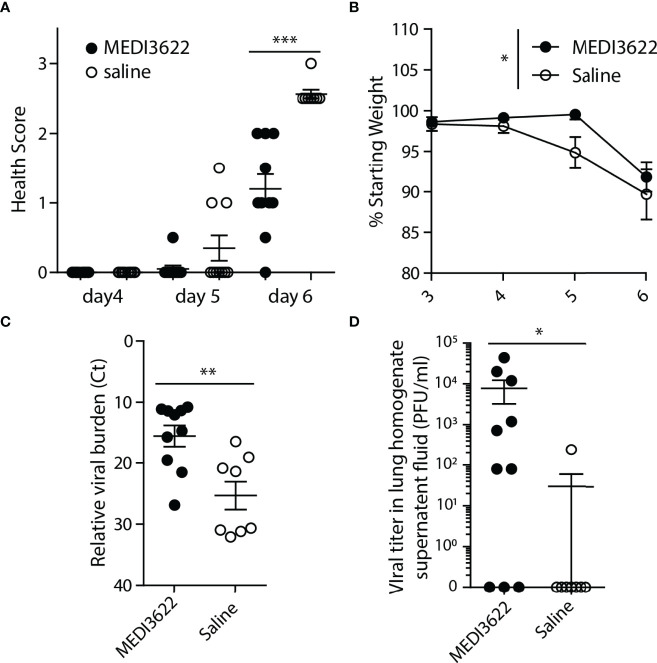
Treatment of SARS-CoV-2 infected mice with MEDI3622 mAb ameliorates morbidity, but increases viral titers. **(A)** MEDI3622 mAb treated mice have diminished morbidity compared to saline-treated mice. **(B)** MEDI3622-treated mice lose significantly less weight than saline-treated controls (represented as the percentage of starting weight at time of infection for each mouse) (2-way ANOVA, *p<0.05). MEDI3622-treated mice have greater relative viral burdens than saline-treated mice as measured by **(C)** qPCR and **(D)** plaque assay Mann-Whitney test *p<0.05, **p<0.01, ***p<0.001. This experiment was performed similarly 2 independent times with 10 mice per group.

ACE2/TMPRSS2/ADAM17 interplay is likely important to SARS-CoV-2 infection and disease (23), though direct evidence for a role of ADAM17 *in vivo* has not yet been established. For these studies, we used the highly specific anti-human and anti-mouse ADAM17 function-blocking mAb MEDI3622 (22, 34–37). We tested the effects of MEDI3622 on disease progression and virus replication in SARS-CoV-2 infected human ACE2 transgenic mice (K18-hACE2) (38). During a 6-day infection, saline-treated mice displayed greater morbidity based on our health scoring system (**Figure 1A**) and two out of 10 of these mice succumbed to infection prior to the end of the 6-day experiment. In contrast, MEDI3622 antibody-treated mice appeared healthier, and all mice survived. Furthermore, saline-treated mice lost significantly more weight than mice in the MEDI3622-treated group (**Figure 1B**). These data indicate that inhibition of ADAM17 activity during SARS-CoV-2 infection reduced morbidity, which is in agreement with a recently proposed model (23). The remaining mice (8 saline-treated and 10 MEDI3622-treated) that survived the duration of the experiment were euthanized and lung viral titers were assessed by qPCR and plaque assay. Viral RNA was detected in all mice, but MEDI3622-treated mice had significantly higher viral burdens in their lungs (**Figure 1C**). Infectious virus was also detectible by plaque assay in the lungs of 7 of 10 MEDI3622-treated mice, but only 1 of 8 surviving saline-treated mice (**Figure 1D**). Thus, despite decreased morbidity and mortality in MEDI3622-treated mice, their lung viral titers exceeded those of saline-treated mice.

**Figure 2:**
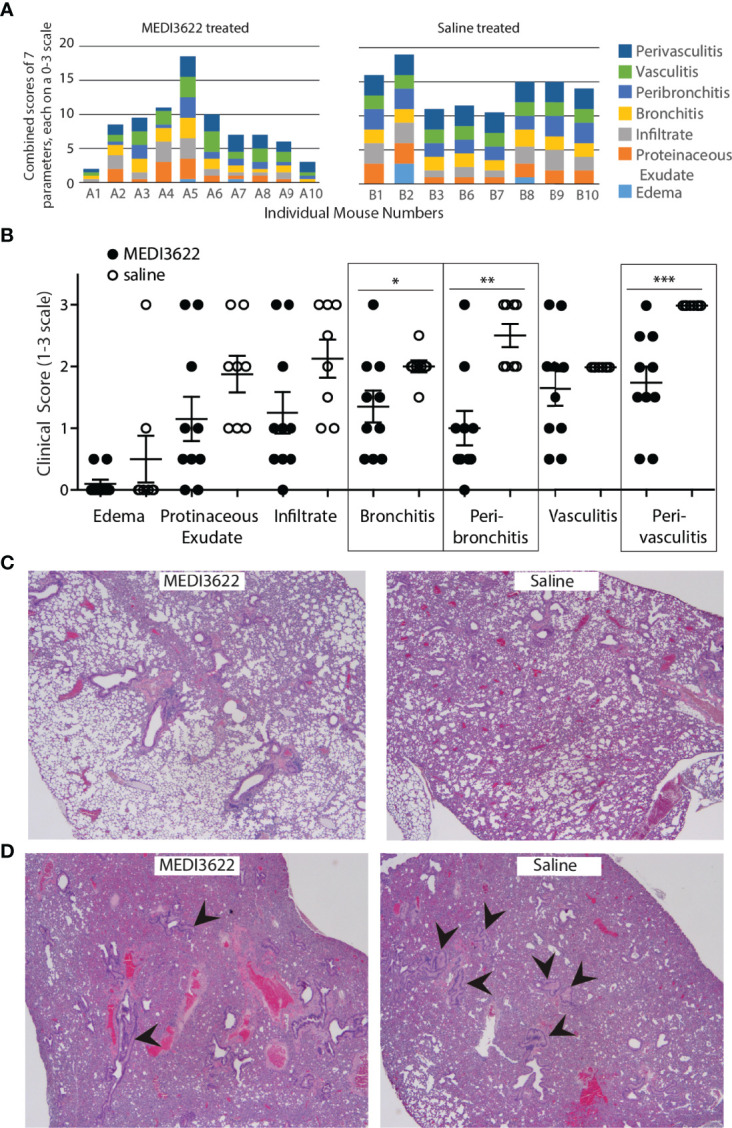
Damage to lungs in MEDI3622-treated SARS-CoV-2 infected mice is significantly reduced compared to those treated with saline. **(A)** Histological scores from parameters shown on right were assessed on a 0-3 scale for each parameter for each mouse. **(B)** Histological scores for each parameter listed in **(A)** are shown. Mann-Whitney test *p<0.05, **p<0.01, ***p<0.001. **(C)** Representative sections from each treatment group show differences in lung damage. **(D)** More mice in the saline-treated group (7 out of 8) had collapsed and fluid filled large bronchi (arrowheads point to examples) than in the MEDI3622-treated group (3 out of 10). This experiment was performed similarly 2 independent times with 10 mice per group. The lung section from the most severely inflamed MEDI3622-treated mouse has fewer collapsed bronchi compared to a representative example from the saline-treated group.

In addition to quantifying virus in the lungs, we also performed histological analyses by assessing damage and inflammation in this tissue. Tissue sections were H&E-stained and scored based on seven unique inflammatory parameters allowing quantification of overall damage. As shown in **Figures 2A, B** the lungs from MEDI3622-treated mice displayed significantly less overall pathology than those from saline-treated mice. There was a significant difference in bronchitis, peribronchitis, and perivasculitis between the groups (**Figure 2B**). Furthermore, representative sections from each group in **Figure 2C** qualitatively support the histological scores noted above. Finally, we observed collapsed large bronchi in the lungs of 7 out of 8 saline-treated mice, and only 3 of 10 MEDI3622-treated mice. Even the most severely affected lung from the MEDI3622-treated group had fewer collapsed bronchi than those represented in the saline-treated group (**Figure 2D**). Thus, the lung histopathology is in strong agreement with greater morbidity and mortality of saline-treated mice compared to those treated with MEDI3622.

**Figure 3:**
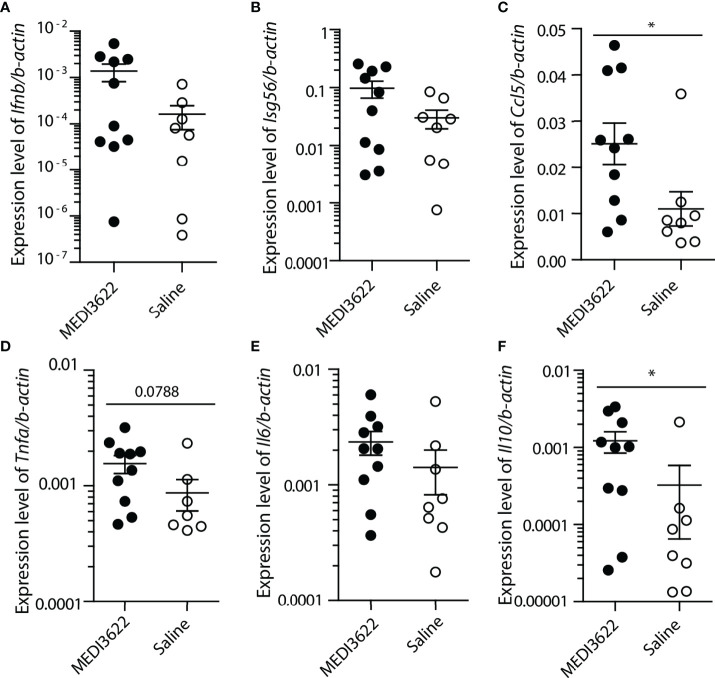
Cytokine transcript levels of MEDI3622-treated mice are higher than or similar to those of control (saline-treated) mice during SARS-CoV-2 infection. RNA was extracted from lungs of mice infected with SARS-CoV-2, described in **Figure 1**. **(A–F)** Transcript levels of genes were measured with quantitative RT-qPCR and normalized against the housekeeping gene β−actin. *p<0.05. This experiment was performed similarly 2 independent times with 10 mice per group.

To quantify changes in pro- and anti-inflammatory gene transcription, we used qPCR to measure relative expression of a number of cytokine and chemokine transcripts in the lung. Interestingly, despite the obvious differences in lung tissue inflammation, there were either no differences between the groups or a slight increase in the MEDI3622-treated mice for most of the transcripts (**Figure 3**). The antiviral transcripts *Ifnb* and downstream *Isg56*, as well as transcripts encoding the chemokine *Ccl5* and inflammatory cytokines *Tnfa* and *Il6* were either slightly increased or there were no differences in lungs of MEDI3622-treated mice, compared to saline-treated mice. *Il10* transcript expression was also increased, which may suggest an anti-inflammatory mechanism. Increased expression of transcripts encoding *Ifnb* and *Isg56* suggest that anti-viral defenses were not compromised in the MEDI3622-treated animals.

**Figure 4:**
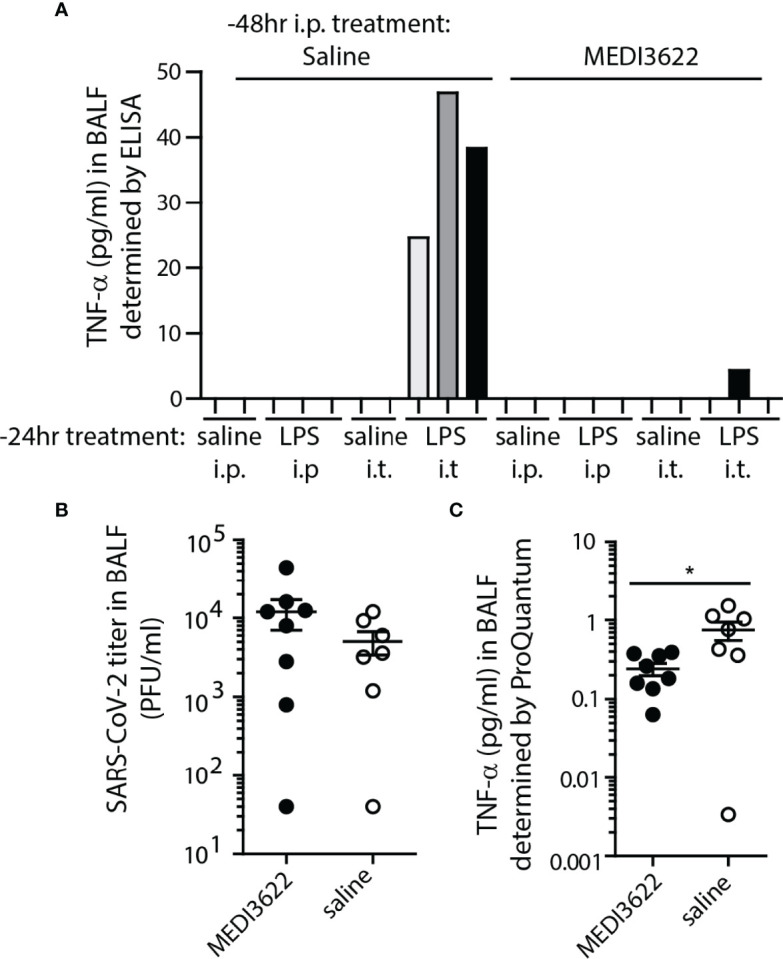
MEDI3622 blocks soluble TNF-α production in the lung. **(A)** Mice were treated 48 hours prior to euthanasia with either saline or MEDI3622 by the i.p. route, then 24 hours later injected with either saline (2 mice per route) or 1ug/mouse *E. coli* LPS (3 mice per route) by either the i.p. or i.t. routes. Only those mice treated i.t. with LPS had TNF-α in BALF 24 hours after injection, and treatment with MEDI3622 blocked this detection. This experiment was performed one time with 2 or 3 mice per group. **(B)** BALFs were collected on day 4 post-infection from SARS-CoV-2 infected, saline or MEDI3622-treated mice. Infectious viral burden in MEDI3622-treated mice was slightly increased at this interval. **(C)** BALF from mice treated with MEDI3622 had significantly less TNF-α compared to control mice. *p<0.05. This experiment was performed one time with 7 or 8 mice per group.

Finally, we demonstrated that MEDI3622 treatment of K18 hACE2-expressing mice decreases soluble TNF-α in the lung. ADAM17 is a sheddase of TNF-α (39, 40). To establish that this occurred in K18 hACE2 mice, they were treated with either MEDI3622 or saline by i.p. injection and LPS or saline delivered either i.p. or i.t. twenty four hours later to stimulate rapid production of inflammatory cytokines, including TNF-α. One day later, the mice were euthanized and BALF was collected. Murine TNF-α was quantified in BALF by ELISA. Only LPS treatment in the lung produced soluble TNF-α detectible in the BALF, but prior treatment with MEDI3266 (delivered i.p.) effectively blocked this production (**Figure 4A**). A similar experiment was performed in mice infected with SARS-CoV-2 for four days, well before onset of symptomatic disease. At this interval, a slight increase in SARS-CoV-2 was already detectible by plaque assay in BALF from MEDI3622-treated mice, but was not significantly different between groups (**Figure 4B**). **Figure 4C** shows that treatment with MEDI3622 resulted in a significant reduction in soluble TNF-α detection. The low values measured in this assay may be explained by the very early preclinical interval for BALF collection. This result is in contrast to *Tnfa* transcripts, which were slightly increased in the lungs of MEDI3622-treated mice (**Figure 3**). Hence, our findings demonstrate that for ADAM17 substrates, an upregulation in their gene expression does not necessarily indicate increased release of their soluble form. The reduction in inflammation and morbidity by MEDI3622 treatment may be due to blocked TNF-α shedding in the lungs of SARS-CoV-2 infected mice.

## Discussion

These data indicate that by blocking ADAM17, the harmful inflammatory effects causing severe disease symptoms are dampened, while viral loads simultaneously increase. Inhibition of ADAM17/MMP with a small molecule inhibitor also reduced lung injury in a non-infectious inflammatory model related to COVID-19 in mice (30). However, since that study did not utilize live virus, viral replication could not be assessed. Furthermore, most small molecule inhibitors inhibit multiple MMPs, but MEDI3622 is highly specific for ADAM17, revealing more definitive evidence of its role. Our results clearly demonstrate the importance of ADAM17 in SARS-CoV-2 induced disease in mice and likely in COVID-19 in humans. The data are consistent with the theoretical model proposed by Zipeto et al., where blocking ADAM17 could diminish damaging inflammation but possibly promote viral replication (23). For clinical relevance, the longer-term effects of MEDI3622 treatment should be assessed. It is possible that healthier lung tissues could eventually overcome viral infection. This result was demonstrated in a study assessing broad-spectrum immunosuppression in MERS-CoV-infected Rhesus Macaques. Immunosuppressed animals eventually overcame higher viral titers in their lungs, but also had some increased spread of the virus to other tissues (41). Potentially, the more specific immunosuppression provided by blocking ADAM17 may retain this benefit at less risk of viral spread.

One probable mechanism underlying the effect of MEDI3622 on lung inflammation is reduction of soluble TNF-α, as is the case in an influenza pneumonia model (42). Reduced TNF-α with MEDI3622 treatment was also shown in the sepsis model (22). In addition, MEDI3622 treatment may have also reduced ACE2 shedding and its soluble levels. Thus, the conversion of angiotensin II to Ang (1–7) by ACE2 is better preserved, serving an immunoregulatory function. MEDI3622 treatment would also leave a greater number of viral receptors on cell surfaces that may enhance viral replication. Interestingly, a recent report suggests sACE2 can facilitate viral uptake into cells and that inhibition of ADAM17 activity greatly suppressed viral replication *in vitro* (31). These findings are difficult to reconcile with our results considering the vast differences in *in vitro* compared to *in vivo* studies. Further detailed investigation will be necessary to determine the effects of MEDI3622 treatment on ACE2 shedding and sACE2 on SARS-CoV-2 infection *in vivo*.

Our findings indicate a key contribution by ADAM17 in inflammation regulation during COVID-19. The need for more and better treatment options for COVID-19 is critical, thus it is important to consider how these data may translate to the clinic. Indeed, MEDI3622, is a human IgG1 antibody and thus amenable to clinical studies. It is feasible that for COVID-19, MEDI3622 could be combined with already approved antiviral drugs to address both the overt inflammation and the high viral load simultaneously. Further exploration and comprehension of this pathway could not only impact treatment of COVID-19 but other viral infections as well, such as influenza. Since strong inflammation in the airways is the cause of pathogenesis in multiple infectious and non-infectious conditions, MEDI3622 could have broad application to address this problem.

## Data Availability Statement

The original contributions presented in the study are included in the article/supplementary material. Further inquiries can be directed to the corresponding author.

## Ethics Statement

The animal study was reviewed and approved by Montana State University Institutional Animal Care and Use Committee.

## Author Contributions

JH, HG, KS, DK, AR-A, BW, and MJ contributed to conception and design of the study. DK, AR-A, and MJ acquired funding for the study. JH, DS, ARo, HG and KB performed the experiments and analyzed the results. JH wrote the first draft of the manuscript while DS, ARo, HG and KB wrote sections of the manuscript. All authors contributed to manuscript revision, read, and approved the submitted version.

## Funding

This work was supported by funding from the MSU Vice-President for Research, Economic Development and Graduate Education office, MSU College of Agriculture and Department of Microbiology and Cell Biology, and USDA/NIFA Multi-State and Hatch Funds.

## Conflict of Interest

The authors declare that the research was conducted in the absence of any commercial or financial relationships that could be construed as a potential conflict of interest.

## Publisher’s Note

All claims expressed in this article are solely those of the authors and do not necessarily represent those of their affiliated organizations, or those of the publisher, the editors and the reviewers. Any product that may be evaluated in this article, or claim that may be made by its manufacturer, is not guaranteed or endorsed by the publisher.
